# A Rare Case of a 54-year-old Male with Vocal Cord Paralysis Secondary to Left Atrial Enlargement

**DOI:** 10.7759/cureus.53463

**Published:** 2024-02-02

**Authors:** Luis E Santiago, Mohammed Alfartusi, Zahid Nadeem, Adesoji Adenigbagbe

**Affiliations:** 1 Internal Medicine, HCA Florida Northwest Hospital, Margate, USA; 2 Internal Medicine, HCA Florida Westside Hospital, Plantation, USA; 3 Critical Care Medicine, HCA Northwest Hospital, Margate, USA

**Keywords:** left atrial enlargement, left recurrent laryngeal nerve palsy, cardiovocal syndrome, pulmonary hypertension, vocal cord paralysis, mitral valve prolapse, ortner syndrome

## Abstract

Ortner's syndrome, a rare condition characterized by hoarseness due to left recurrent laryngeal nerve palsy caused by cardiovascular structural compression, is typically associated with an enlarged left atrium secondary to conditions like mitral stenosis. However, recent studies propose additional causes, including compression between the dilated pulmonary artery and the aorta. We present a case of a 54-year-old male with Ortner's syndrome secondary to severe mitral regurgitation and pulmonary hypertension. Our patient presented with a one-month history of progressive dyspnea and hoarseness. Diagnostic imaging revealed cardiac enlargement, left vocal cord paralysis, and severe mitral valve pathology. A transesophageal echocardiogram revealed mitral valve prolapse and severe flail motion of the anterior leaflet.

Further assessments through catheterizations confirmed severely elevated right ventricular systolic pressures and pulmonary hypertension. Attempts at mitral valve replacement were hindered by persistently elevated pulmonary pressures, necessitating transfer for specialized care. Our case highlights the broad differentials for hoarseness, emphasizing rare cardiovascular origins such as Ortner's syndrome, involving compression of the left recurrent laryngeal nerve. Early identification is essential, often necessitating comprehensive head and neck examination and radiological studies. While management depends on nerve injury duration, a timely intervention targeting the underlying cardiovascular pathology, including appropriate medical therapy and surgical approaches, can potentially alleviate or reverse nerve damage. Furthermore, our case underscores the significance of initiating guideline-directed medical therapy early in chronic cardiovascular conditions to mitigate cardiac remodeling and prevent complications like left recurrent laryngeal nerve palsy. Timely identification and targeted management of underlying cardiovascular etiologies are crucial in preventing Ortner's syndrome.

## Introduction

Ortner's syndrome (OS), named after Nobert Ortner, who first described the phenomenon in 1897, involves hoarseness resulting from left recurrent laryngeal nerve (RLN) palsy due to mechanical compression by adjacent cardiovascular structures. Previously, the primary cause was often attributed to an enlarged left atrium from conditions like mitral stenosis [1]. However, recent studies suggest that nerve palsy can be caused by compression between the dilated pulmonary artery and the aorta, such as in thoracic aortic aneurysms or pulmonary hypertension, which have emerged as the primary etiologies [2-3].

The syndrome's typical presentation consists of hoarseness, which may be accompanied by signs of heart failure, such as volume overload. These patients tend to experience a gradual development of hoarseness, which can progress to complete loss of voice depending on the severity of the injury to the left recurrent laryngeal nerve [3]. 

Various clinical conditions can cause recurrent laryngeal nerve palsy; some of these conditions are pulmonary hypertension, Eisenmenger's syndrome, mitral regurgitation, patent ductus arteriosus, left ventricular aneurysm, aortic pseudoaneurysms, aortic intramural hematomas, and mitral stenosis [3]. A prospective study by Loughran Et al. reported the incidence of left RLN palsy due to OS to be 11% [4]. The most common etiology of OS is aortic aneurysms (41%), while OS due to left atrium enlargement makes up 27% of cases [3]. Diagnostic evaluations might involve transthoracic echocardiography (TTE), chest radiography, CT, MRI, and laryngoscopy to confirm left vocal cord paralysis [3]. Treatment depends on the underlying cause and may include thoracic surgery, radiation therapy, endovascular aortic repair, or appropriate guideline-directed medical therapy (GDMT) for heart failure [3].

Ortner's syndrome cases are rare in the literature [3]; they show the importance of clinical presentation and history in guiding diagnostic investigations. We present a case of a 54-year-old male with atrial enlargement secondary to severe mitral regurgitation (MR) and severe pulmonary hypertension presenting as Ortner's syndrome.

## Case presentation

**Figure 1:**
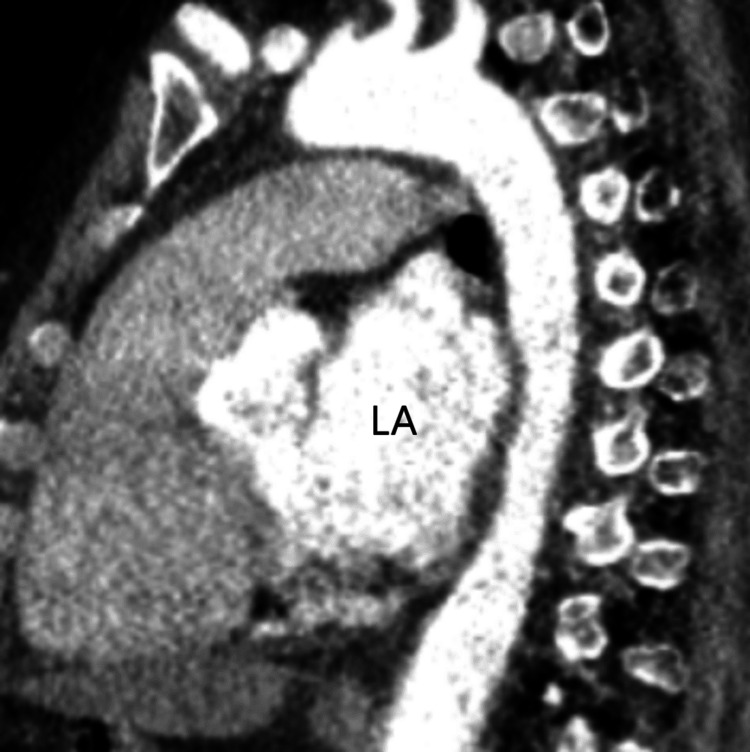
Sagittal computed tomography of the chest showing marked left atrium enlargement. LA: Left Atrium

**Figure 2:**
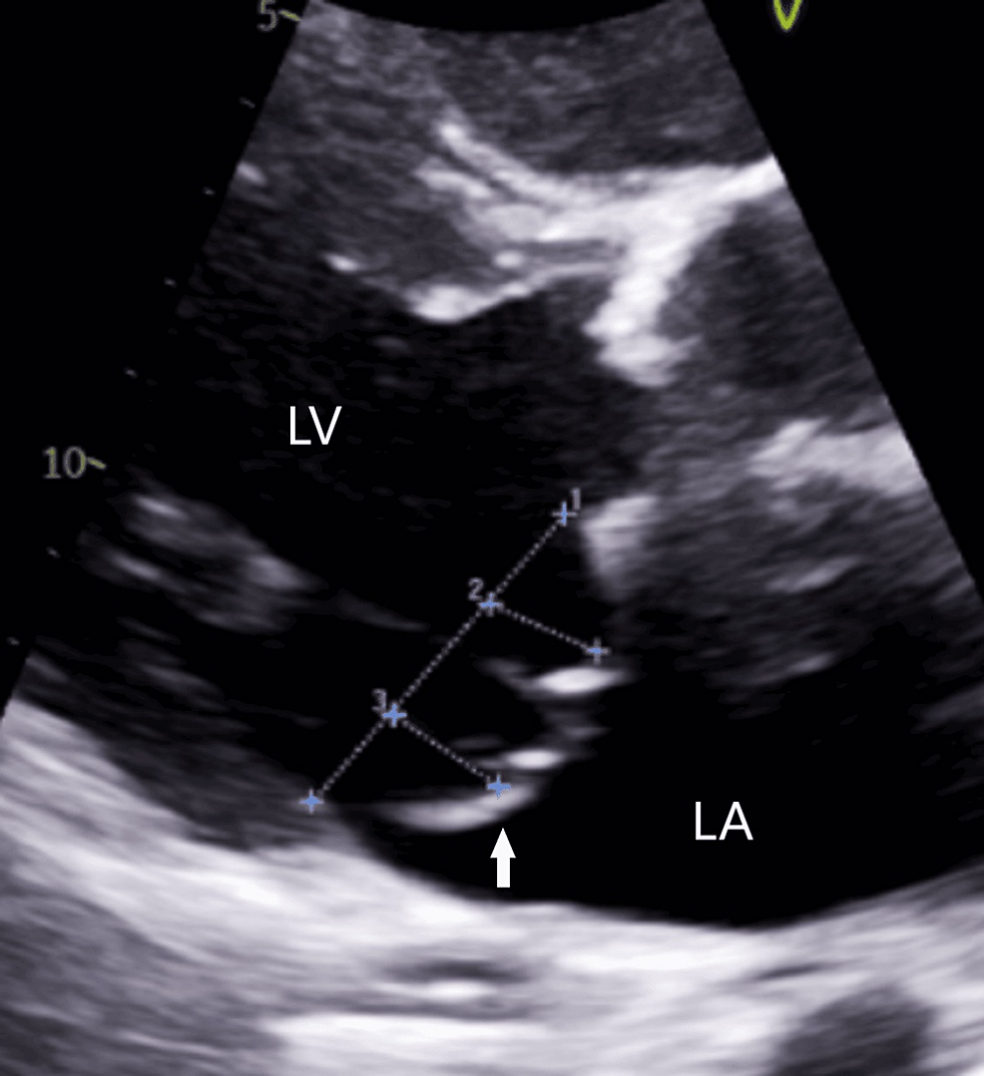
Transthoracic echocardiogram parasternal view showing mitral valve prolapse (arrow). The mitral valve opening was 3.9 cm (1). The mitral valve prolapse was 1.2 cm (2) and 1.3 cm (3) LA: Left Atrium; LV: Left Ventricle

A 54-year-old male with a past medical history of severe mitral regurgitation presented to the emergency department with a one-month history of progressing dyspnea and hoarseness. The patient denied any history of chest pain, fever, or hemoptysis. On arrival, the patient was afebrile; blood pressure was 100/67 mm Hg; the heart rhythm was rapid and irregular; the oxygen saturation was 94% on room air. Auscultation revealed crackles and a decrescendo, holosystolic murmur at the apex radiating to both the precordium and the axilla. Jugular venous distension was present. An electrocardiogram showed atrial fibrillation with a fast ventricular response. Computed tomography of the chest showed cardiac silhouette enlargement (Figure 1). A transthoracic echocardiogram revealed a marked mitral valve prolapse with wide-open regurgitation (Figure 2). A neck CT revealed paramedian positioning of the left true vocal cord, which suggests left vocal cord paralysis. Laboratory values, including inflammation markers, were normal.

A laryngoscopy was performed for further evaluation, which confirmed the left vocal cord paralysis. The left vocal cord was in midline position and slightly shortened. No mobility was visualized on the left vocal cord. 

**Figure 3:**
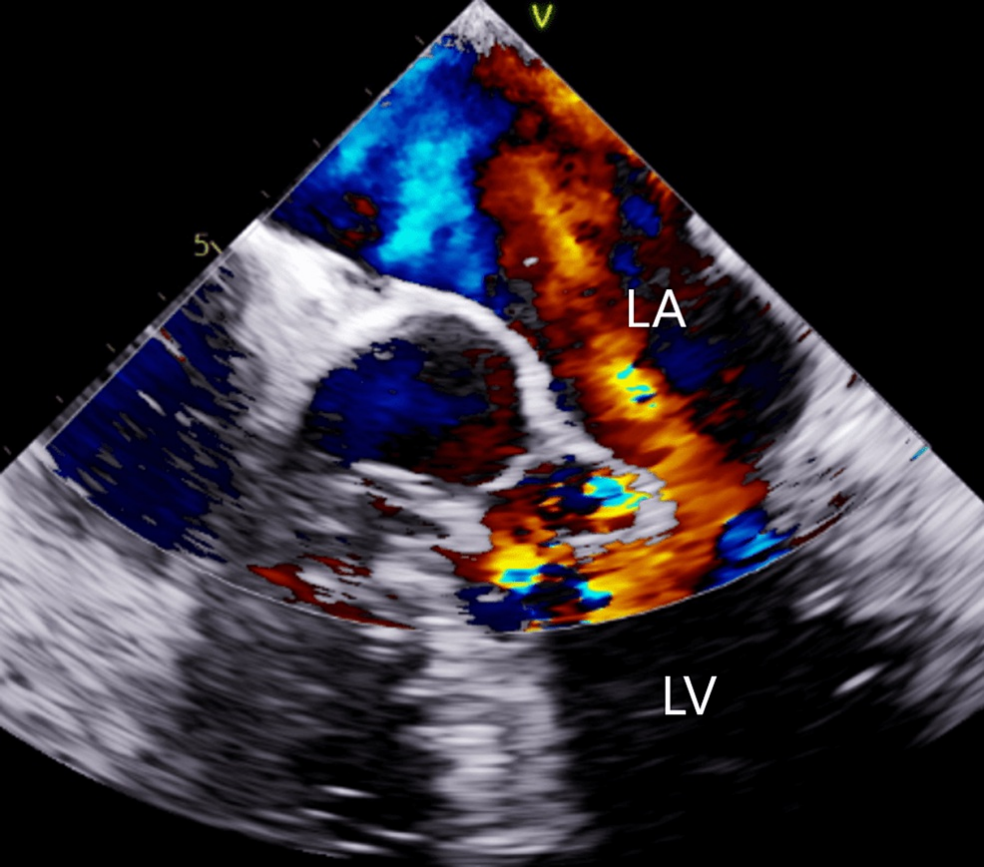
Transesophageal echocardiogram mid-esophageal 4-chamber view showing mitral valve prolapse with wide-open regurgitation (red area). LA: Left Atrium; LV: Left ventricle

On day two of admission, the patient had a left heart catheterization (LHC), and right heart catheterizations performed (RHC), which were significant for elevated left and right filling pressures, pre and post-capillary pulmonary hypertension, severe mitral regurgitation, and normal coronary arteries. Ejection fraction was 50-55%, mean arterial pressure was 77 mmHg, and left ventricular end-diastolic pressure was 20 mmHg. The right atrial mean pressure was 12 mmHg, right ventricular end-diastolic pressure was 14 mmHg, mean pulmonary capillary wedge pressure was 49 mmHg, and pulmonary artery pressure was 95/47 mmHg. Hours later, the patient was noted to be hypotensive with a blood pressure of 85/53 mmHg. Secondary to the patient's hypotension and elevated filling pressures, the decision was then made for an intra-aortic balloon pump placement (IABP), and a furosemide drip was started. A transesophageal echocardiogram (TEE) was performed, which showed a mitral valve prolapse and severe flail motion of the anterior leaflet. There was wide-open regurgitation (Figure 3). The subsequent timeline of events is noted in Table 1.

**Table 1:** Timeline of events MVR: Mitral Valve Replacement

Day	Event
3	Intra-aortic balloon pump (IABP) removal
5	Pulmonary artery (PA) pressure: 90/40 mmHg. Milrinone and Sildenafil were initiated
8	Pulmonary pressures measured by Swan-Ganz catheter improved: 46/34 mmHg
13	The patient was taken for MVR, but surgery was canceled due to elevated PA pressure
14	Transferred to a tertiary care center for mitral valve replacement

## Discussion

The differential diagnosis of hoarseness is broad; these include inflammatory etiologies (50% of cases), neuromuscular and psychiatric disorders (2.8%-8%), malignancy (2%-3%), and other causes such as amyloidosis and hypothyroidism [5]. In rare cases, hoarseness can be caused by an underlying cardiovascular condition known as Ortner's syndrome. Given the rarity of this syndrome, it is usually not considered in the initial differential diagnosis of hoarseness.

The left recurrent laryngeal nerve comes from the left vagus nerve, runs across the aortic arch, loops back around the ligamentum arteriosum, and then travels up the tracheoesophageal groove. Due to its course, it is susceptible to compression by nearby structures [6]. These nerves are responsible for providing all intrinsic muscles of the larynx except for the cricothyroid muscles [5]. Initially, it was believed that the enlarged left atrium directly compressed the left RLN nerve [1]. However, recent studies by Paquette Et al. suggested that the dilated left atrium pushes the left pulmonary artery toward the aorta, subsequently compressing the left RLN between the left pulmonary artery and aortic arch, leading to ischemic injury and degeneration of the nerve fibers [7].

Early diagnosis of the etiology of the left recurrent laryngeal nerve palsy is important as prompt treatment can reverse the nerve damage in certain cases, depending on the duration. Patients who experience hoarseness lasting more than two weeks and do not have symptoms of acute respiratory infection should undergo a complete head and neck examination, including visualization of the laryngopharynx, by an otorhinolaryngologist. Computed tomography scanning can add valuable information [8]. Radiological studies are essential for ruling out any masses that may be compressing the nerve. Chest radiographs are usually obtained first, followed by CT imaging. CT is particularly helpful for inspecting the aortopulmonary region, which may be missed on a chest x-ray. In our case, a laryngoscopy was performed, which showed left vocal cord paralysis. A neck CT showed a paramedian position of the left true vocal cord, which is suggestive of left vocal cord paralysis.

Treatment of Ortner's syndrome depends on the duration of the injury. In a review of 117 patient cases with Ortner's syndrome by Verma Et al., it was noted that there was an improvement in hoarseness in 85.4% of patients managed with surgical intervention. Open aortic or thoracic endovascular aortic repair (TEVAR) was the most common treatment modality in patients with OS due to aortic aneurysm. In patients with congenital heart disease, such as atrial septal defect (ASD) or ventricular septal defect (VSD), surgical closure of these abnormalities was performed. Angioplasty, thyroplasty, and medialization of the vocal cords were some other procedures performed [3]. Another review of three cases with OS by Hong Et al. reported improvement in hoarseness in all three cases treated with injection laryngoplasty [9].

## Conclusions

Our case report details a 54-year-old male with Ortner's syndrome secondary to severe mitral regurgitation and pulmonary hypertension, emphasizing the rarity of this syndrome in the literature. Despite attempts at mitral valve replacement being impeded by persistently elevated pulmonary pressures, early identification of the syndrome's cardiovascular origins remains essential.

This case highlights the complexity of differential diagnoses for hoarseness and the significance of recognizing rare cardiovascular etiologies such as Ortner's syndrome. The pathophysiology involving compression of the left recurrent laryngeal nerve between cardiovascular structures elucidates the need for prompt identification through comprehensive head and neck examinations and radiological studies. Furthermore, our case underscores the importance of timely identification and targeted management of underlying cardiovascular conditions to prevent the occurrence of Ortner's syndrome. Initiating guideline-directed medical therapy early in chronic cardiovascular diseases may mitigate complications such as left recurrent laryngeal nerve palsy, highlighting the critical role of preventive cardiovascular care in averting rare syndromes like Ortner's.
